# Tumor immunogenomic signatures improve a prognostic model of melanoma survival

**DOI:** 10.1186/s12967-021-02738-0

**Published:** 2021-02-17

**Authors:** Leah Morales, Danny Simpson, Robert Ferguson, John Cadley, Eduardo Esteva, Kelsey Monson, Vylyny Chat, Carlos Martinez, Jeffrey Weber, Iman Osman, Tomas Kirchhoff

**Affiliations:** 1 Laura and Isaac Perlmutter Cancer Center, NYU Langone Health, 522 First Avenue, New York City, NY 10016 USA; 2 Departments of Population Health and Environmental Medicine, NYU Langone Health, New York, USA; 3 The Interdisciplinary Melanoma Cooperative Group, NYU Langone Health, New York, USA; 4 Department of Medicine, NYU Langone Health, New York, USA; 5 Ronald O. Perelman Department of Dermatology, NYU Langone Health, New York, USA

**Keywords:** Melanoma, Genomics, Immunology, Mutation burden, Survival

## Abstract

**Background:**

Tumor mutation burden (TMB) has been associated with melanoma immunotherapy (IT) outcomes, including survival. We explored whether combining TMB with immunogenomic signatures recently identified by The Cancer Genome Atlas (TCGA) can refine melanoma prognostic models of overall survival (OS) in patients not treated by IT.

**Methods:**

Cox proportional-hazards (Cox PH) analysis was performed on 278 metastatic melanomas from TCGA not treated by IT. In a discovery and two validation cohorts Cox PH models assessed the interaction between TMB and 53 melanoma immunogenomic features to refine prediction of melanoma OS.

**Results:**

Interferon-γ response (IFNγRes) and macrophage regulation gene signatures (MacReg) combined with TMB significantly associated with OS (p = 8.80E−14). We observed that patients with high TMB, high IFNγRes and high MacReg had significantly better OS compared to high TMB, low IFNγRes and low MacReg (HR = 2.8, p = 3.55E−08). This association was not observed in low TMB patients.

**Conclusions:**

We report a model combining TMB and tumor immune features that significantly improves prediction of melanoma OS, independent of IT. Our analysis revealed that patients with high TMB, high levels of IFNγRes and MacReg had significantly more favorable OS compared to high TMB patients with low IFNγRes and low MacReg. These findings may substantially improve current melanoma prognostic models.

## Background

Immune checkpoint inhibition (ICI) treatments have substantially improved survival in patients with metastatic melanoma [1, 2], one of the most immunogenic tumors. While the mechanisms of melanoma immunogenicity are not fully understood, observations from ICI treatments suggest that host immune control of the tumor progression is mediated by immune reactivity to neo-epitopes resulting from increased tumor mutation burden (TMB) [3, 4]. It has been established that the immunogenicity of melanoma is driven by one of the highest somatic mutation rates among all cancers [5, 6], and a high overall TMB is associated with improved ICI therapy outcomes in melanoma [7], non-small cell lung cancer (NSCLC) [3], and other cancers [8–10]. Since ICI therapies boost immune priming and activation, the level of neo-epitope burden (NB) resulting from tumor-specific somatic mutations may be associated with improved immune detection of tumor cells. This suggests that the host immune system, depending on tumor properties, has the capacity to control tumor progression from early stages to metastatic presentation, hence impacting overall survival (OS). However, TMB/NB alone does not capture the observed heterogeneity of ICI-related outcomes or OS, suggesting that other modifying factors are involved. Recently, a large study by The Pan-Cancer Immune Working Group, derived from The Cancer Genome Atlas (TCGA) data, comprising 11,080 tumor samples, has classified common cancers into six immune subtypes, grouped by specific immune signatures [11]. The six immunogenomic subtypes were associated with different cancer properties, such as proliferation or survival. These pan-cancer immune signatures provide broad evidence of the contributing role of the tumor immune landscape in cancer progression. Recent evidence has also shown that the immune microenvironment can be associated with cancer specific survival [12, 13]. In melanoma, however, ~ 80% of the tumors from the TCGA analyzed in The Pan-Cancer Immune Working Group study [11] were not classified into any of the six immune subtypes identified in pan-cancer analysis, potentially due to the vast majority of the 80% “unclassified” fraction of the melanoma cohort in TCGA being metastatic. Given that only a small fraction of available samples in melanoma TCGA has been classified, the broader impact of immune/tumor properties on melanoma specific cancer outcomes remains unclear. This strongly implies that the development of multivariable models combining immune features from pan-cancer analysis, with genomic and clinical markers is clinically needed to refine and improve current melanoma-specific prognostic algorithms.

In this report, we tested the hypothesis that the TMB in metastatic tumors predicts OS in melanoma patients not treated by ICI. Using the data from metastatic tumor whole-exome sequencing (WXS), and recently published data on the melanoma immune landscape, both collected as part of TCGA, we aimed to develop a composite biomarker that can substantially improve prognostic models of melanoma survival. We examined whether TMB in metastatic tumors retrospectively predicts OS from the time of primary melanoma diagnosis, and whether such TMB prognostic power could be further refined by inclusion of other recently collected and characterized melanoma immunogenomic tumor features from TCGA [11].

## Materials and methods

### Study population

Using the Genomic Data Commons Data Portal (GDCDP) we accessed The Cancer Genome Atlas (TCGA) skin cutaneous melanoma (SKCM) project [14], and identified 278 metastatic cutaneous melanoma patients with sufficient clinical follow-up from primary diagnosis, who had not been treated with immune-checkpoint inhibition therapies (ICI) (either anti-CTLA-4 or anti-PD-1) at the time of death or last follow-up. Clinical and demographic information including sex, age at diagnosis, age at tumor biospecimen accession, stage at primary diagnosis, overall survival (OS) time, post-accession survival time, and somatic variants called from WXS data of tumor tissues aligned to human reference genome GRCh38 were available from TCGA. Additional information, including gene expression profiles and histological data, were provided by Thorsson et al. [11]. Combining these data, we evaluated 56 features, including somatic mutation data, immunogenomic signatures and tumor microenvironment (TME) characteristics (the data features analyzed and their source are summarized in Additional file 1: Table S1). Using R v.3.5.1., we randomly sampled the patient population into discovery (N = 139, 50% of total sample population) and two validation groups, validation 1 (N = 70, 25%) and validation 2 (N = 69, 25%) (Additional file 1: Table S2).

### HLA-typing by POLYSOLVER

In order to predict which neo-epitopes, derived from somatic mutations, would be presented by MHC class I, and hence may represent cancer-specific antigens, we inferred the major MHC class I alleles (for HLA-A, HLA-B, and HLA-C) for each sample from WXS. For this purpose, we used POLYSOLVER [15], which was developed as part of a pipeline to detect somatic mutations in class 1 HLA genes, a task that is confounded by the high degree of polymorphism present in HLA loci. The POLYSOLVER program has been described extensively elsewhere [15]; briefly, it aims to identify the HLA type from low-coverage WXS by aligning reads to a set of known HLA alleles, then performing a Bayesian calculation incorporating base quality scores, insert sizes, and population-specific allele frequencies. After this processing, POLYSOLVER reports the two most likely alleles for each of HLA-A, HLA-B, and HLA-C. After updating POLYSOLVER source code for compatibility with the human reference genome GRCh38.p12 and SAMtools v1.8 [16], blood-derived normal BAM files for each sample were used as inputs to POLYSOLVER under default parameters. The four-digit names for the two most likely alleles for each of HLA-A, HLA-B, and HLA-C were retained for determination of neo-epitope burden.

### Quantification of mutation and neo-epitope burden

Using the MuTect2 aggregated protected MAF file, TMB for each patient was calculated as the total number of non-silent mutations present on autosomal chromosomes (frameshift or in-frame indels, missense mutations, nonsense mutations, nonstop mutations, or mutations in RNA-splice or transcription start sites). To define neo-epitope burden (NB), all amino acid 9-mers incorporating non-silent mutations from autosomal chromosomes were taken as candidate neo-epitopes and used as input to netMHC 4.0 [17] in conjunction with patient HLA types identified by POLYSOLVER. Using a neural network approach, netMHC 4.0 measures the binding affinity between a peptide and an associated HLA type. Those candidate neo-epitopes receiving a “top 1%” affinity score from netMHC 4.0 were defined as neo-epitopes, and a patient’s associated neo-epitope burden was defined as the total number of neo-epitopes present. Pearson correlation coefficients were determined to assess the relationship between the TMB and NB scores.

### Statistical analysis

Survival analyses were performed using Cox PH models to determine associations of immunogenomic and TME characteristics (Additional file 1: Table S1) [11] with OS in patients. All models were adjusted by age and stage at primary diagnosis, defined by American Joint Committee for Cancer (AJCC) as prognostic predictors. For each metastatic tumor analyzed in this study, we used the AJCC staging data of primary diagnosis defined as local (stage I/II) or regional/advanced (stage III/IV). The grouping into two categories was to increase statistical power due to limited number of stage IV patients at primary diagnosis (N = 14) and the disproportionate number of patients identified as stage III (N = 127) compared to those who were stage I (N = 67) or stage II (N = 70) at primary diagnosis.

To define high and low TMB, various thresholds of total number of non-silent somatic mutations per exome per patient from TCGA SKCM WXS (50, 75, 100, 125, 150, 200, 250, and the median mutation burden of 323) were tested to dichotomize patients in the discovery set (N = 139) by TMB. The threshold that resulted in the most significant association of TMB with OS in a univariate Cox PH model was considered optimal. The eight potential TMB thresholds were then tested via univariate Cox PH analyses on 1000 bootstrapped iterations of the discovery set data to ensure the optimal threshold was robust to sampling variation. Once this threshold was established (N = 125 mutations), the validation 1 group (N = 70) was used to define low and high TMB. Similarly, for the NB analysis, thresholds of 25, 50, 75, 100, 125, 150, and 189 (median NB value) mutations per exome per patient were evaluated in the discovery data (N = 139) and confirmed on 1000 bootstrapped iterations; the optimal threshold was moved forward to validation 1 (N = 70). Following validation, all samples were aggregated to build final Cox PH models.

Univariate Cox PH models were built on the discovery data set, tumor mutation burden (low [TMB ≤ 125 mutations], high [TMB > 125 mutations]), and 53 immune expression signatures, genomic features and factors of TME. Significant associations (p-value < 0.05) were then tested in the validation 1 data set. Features that were significantly associated with survival in both the discovery and validation 1 sets and which showed no statistically significant difference in distribution between the sets were moved forward for inclusion in a multivariate Cox PH model. For all possible permutations of those covariates, multivariate Cox PH models were built using the discovery data set. To ensure the independent validation of these features from univariate analysis, in multivariate Cox PH models an independent validation set (validation 2 data set, N = 69) was used. After identifying the optimal multivariate model, the proportional hazard assumption was tested for all included covariates by testing for independence between the scaled Schoenfeld residuals [18] for that covariate and time, with independence indicating that the proportional hazard assumption was valid.

To test the association of the selected covariates on OS conditioned on TMB status, the total patient population was divided into two subsets: one comprising patients with high TMB (> 125) (N = 220) and the other comprising patients with low TMB (≤ 125) (N = 58). The multivariate model constructed from the full analysis was replicated in each subset to determine how the covariates affect OS within each set. To assess the combined effect of the levels of IFNγRes and MacReg in these subsets, Cox PH regressions were modeled using continuous values for the MacReg and IFNγRes signatures. Kaplan–Meier (KM) curves were then drawn for high and low IFNγRes and MacReg levels, dichotomized by their respective total population medians, in each of the high TMB and low TMB data sets. For the KM analyses, patients with high MacReg (> overall median) and high IFNγRes (> overall median) values were coded as 1, and those with low values for both (≤ overall median) were coded as 0. A multivariate Cox PH regression was also performed on all patients with available survival data from the time point of advanced melanoma tissue acquisition and accession (“post-accession survival”). For all patients with post-accession survival data (N = 195) available, Cox PH models were developed including TMB and immunogenomic features identified in OS model, adjusting for sex and age at tumor accession. All analyses were performed using R v3.5.1 and packages ‘survival’ (v.2.43.3) [19, 20] and ‘survminer’ (v.0.4.3) [21]. Wald test p-values are reported for univariate Cox PH analyses and log-rank p-values are reported for multivariate Cox PH models unless otherwise indicated.

## Results

In the publicly available data from TCGA [14], we identified 278 metastatic melanoma patients who had not been treated with ICI. These patients were randomly divided into discovery (N = 139, 50%) and two validation sets (validation 1 N = 70, 25% and validation 2 N = 69, 25%), to ensure reproducibility of the findings. We calculated TMB for each tumor sample in the discovery and validation sets. Using the patient samples in the discovery set, we tested TMB as a prognostic marker for OS by stratifying patients into low and high TMB according to the number of non-silent somatic mutations per exome per patient present in their tumor sample. The median TMB across samples in the discovery set was 323 mutations (range 12–14,548). To define TMB cut-off most significantly associated with OS, in the discovery set we tested thresholds of 50, 75, 100, 125, 150, 200, 250 mutations per exome per patient (Table 1). We also tested a threshold of 323 mutations (median TMB across samples in the discovery set). A threshold of 125 mutations used to define low vs high TMB was found most significantly associated with OS, with a univariate Cox PH p = 1.30E−05, showing that patients with TMB of ≤ 125 mutations have significantly shorter median OS (2.4 years, p = 1.30E−05, HR = 3.52, 95% CI 2.00–6.20) compared to those with TMB of > 125 mutations (9.3 years) (Table 1 and Additional file 1: Figure S1A). The 125 TMB threshold was also the optimal TMB cut-off when tested on 1000 bootstrapped iterations of the discovery data (data not shown). We then used 125 mutations as our cutoff in the validation 1 set and observed that patients with 125 or fewer mutations had significantly worse median OS (3.6 years, p = 0.01, HR = 2.86, 95% CI 1.23–6.62) when compared to those with more than 125 mutations (9.8 years) (Table 1 and Additional file 1: Figure S1B). The pooled-analysis combining the discovery and validation 1 subsets showed the most significant association with OS (p = 4.01E−07, HR = 3.36, 95% CI 2.10–5.36, Table 1 and Additional file 1: Figure S1C).

**Table 1:** The different thresholds of the number of somatic mutations used to define high and low TMB, the corresponding number of low TMB patients per each threshold (N < Threshold) in the discovery phase, and associations with OS in discovery, validation, and pooled cohorts

TMB Threshold	N < Threshold	Discovery (N = 139)		Validation 1 (N = 70)		Pooled (Discovery + Validation 1) (N = 209)	
		P	HR (95% CI)	P	HR (95% CI)	P	HR (95% CI)
50	17 (12.2%)	8.64E−05	4.28 (2.07–8.84)				
75	23 (16.5%)	4.57E−05	3.69 (1.97–6.92)				
100	28 (20.1%)	1.11E−04	3.18 (1.77–5.71)				
*125*	*34 (24.5%)*	*1.30E−05*	*3.52 (2.00–6.20)*	*0.01*	*2.86 (1.23–6.62)*	*4.01E−07*	*3.36 (2.10–5.36)*
150	36 (25.9%)	3.36E−05	3.15 (1.83–5.41)				
175	40 (28.8%)	2.24E−04	2.78 (1.62–4.78)				
200	46 (33.1%)	2.22E−03	2.28 (1.34–3.86)				
323	71 (51.1%)	8.69E−02	1.59 (0.94–2.70)				

The p-values (P), hazard ratios and 95% confidence intervals (HR 95% CI) for each threshold in the discovery phase were derived from univariate Cox PH models. The 125-mutation threshold (italic), which had the most significant p-value in the discovery phase, was moved forward to the validation stage. The p-value (P), hazard ratio, and 95% confidence interval (HR 95% CI) from the univariate Cox PH model from the validation phase are also included. The final two columns present the p-value (P) from the meta-analysis of the entire population, with accompanying hazard ratio and 95% confidence interval (HR 95% CI) from a univariate Cox PH model

From the total TMB, the neo-epitope burden (NB) for each sample was determined by examining the binding affinity of potential amino acid 9-mers induced by non-silent somatic mutations depending on the HLA type. The median NB across samples in the discovery set was 189 neo-epitopes (range 1–10,245), and we found the two to be highly correlated (Pearson’s r = 0.979, p < 1E−20, Additional file 1: Figure S2). We then tested NB as a prognostic marker for OS. Using the discovery set, we stratified patients into low or high NB using thresholds of 50, 75, 100, 125 and 150 neo-epitopes (Additional file 1: Table S3). A threshold of 189 (median NB across samples in discovery set) was also tested. Stratifying the patient groups by 50 NB threshold showed the most significant associations with OS (p = 3.84E−05, HR = 3.50, 95% CI 1.93–6.35) in the discovery set (Additional file 1: Figure S1D); patients with low NB (≤ 50 neo-epitopes) have significantly worse OS (median OS = 2.4 years) compared to those with high NB (> 50 neo-epitopes, median OS = 9.3 years). This threshold was found to be optimal in the bootstrap analysis and was moved forward to the validation 1 phase: while low NB showed comparable effect to low TMB (low NB = 3.6 years, high NB = 9.8 years) (Additional file 1: Figure S1B and E, Table 1, Additional file 1: Table S3) the association with OS did not reach statistical significance in validation 1 cohort (p = 0.165, HR = 1.78, 95% CI 0.79–4.01). However, the pooled-analysis of discovery and validation 1 sets, defining low NB as those with fewer than 50 neo-epitopes, was statistically significant (p = 5.60E-05, HR = 2.64, 95% CI 1.65–4.23, Additional file 1: Table S3, Figure S1F). When comparing the samples defined as low TMB (threshold ≤ 125 mutations) and those defined as low NB (threshold ≤ 50 neo-epitopes), there was almost a complete overlap, as expected by the high correlation between TMB and NB; only 14 patient samples were classified as low TMB and high NB, or vice versa. Hence, while there was a clear correlation between TMB and NB, TMB showed superior prognostic ability to predict OS and as such was considered in further analyses.

To refine the TMB association with OS, we have examined other factors of tumor immune microenvironment potentially contributing to the modulation of melanoma OS in patients that did not receive ICI. The goal was to generate a comprehensive multivariate survival model improving the predictive ability of TMB for OS, by testing 53 immunogenomic features previously associated with immune phenotypes in a pan-cancer analysis [11]. First, we constructed univariate Cox PH models for each variable separately in the discovery set, including TMB. As shown in Table 2, seven immunogenomic variables significantly associated with OS in the discovery set (N = 139) and these were tested in validation 1 set (N = 70). Of these, four variables were validated for association with OS in univariate analysis: TMB (discovery: p = 1.30E−05, HR = 3.52, 95% CI 2.00–6.20; validation 1: p = 0.01, HR = 2.86, 95% CI 1.23–6.62), IFNγRes (continuous) (discovery: p = 1.43E−03, HR = 0.63, 95% CI 0.48–0.84; validation 1: p = 8.01E−03, HR = 0.47, 95% CI 0.27–0.82), MacReg (continuous) (discovery: p = 8.18E−03, HR = 0.62, 95% CI 0.43–0.88; validation 1: p = 0.02, HR = 0.56, 95% CI 0.35–0.89) and Lymphocyte infiltration signature score (continuous) (discovery: p = 2.29E−02, HR = 0.80, 95% CI 0.67–0.97; validation 1: p = 0.04, HR = 0.78, 95% CI 0.62–0.99). The four features that passed validation stage were subsequently considered for the final multivariate model. In discovery cohort (N = 139) we tested all possible combinations of the four tumor-specific variables validated in univariate Cox PH analysis (IFNγRes, MacReg, Lymphocyte infiltration signature score, and TMB) as covariates in multivariate Cox PH models of OS (Table 3). These resulted in 51 models tested, each adjusted by stage at diagnosis and age at diagnosis. To achieve the best possible reproducibility, these associations have been validated in an additional independent cohort of N = 69 patients (“validation 2”, see Materials and Methods). Finally, we performed a pooled multivariate Cox PH model (discovery + validation 1 + validation 2). The best fit model (assessed by log-rank test) with the following covariates achieved the strongest statistical significance: TMB, IFNγRes, MacReg, age at diagnosis, and stage at diagnosis (Table 3). Of note, the validity of these findings is supported by incrementally increasing significance of the associations observed with the addition of each validation stage: log-rank p = 1.70E−07 (discovery), pooled log-rank p = 9.00E−10 (discovery + validation 2), and meta-analysis log-rank p = 8.80E−14 (discovery + validation 1 + validation 2). To test how individual covariates contribute to this model, we present the hazard ratios and p-value for each variable separately in this multivariate Cox PH analysis in Table 4. The Cox PH assumption was evaluated for this model by testing for independence between the scaled Schoenfeld residuals for each covariate and time, which showed that the assumption was valid (Additional file 1: Table S4). Interestingly, TMB showed a largest individual effect of all covariates in both univariate (discovery + validation 1 h = 3.36) and multivariate meta-analysis (discovery + validation 1 + validation 2) (HR = 2.56) when compared to age and stage at diagnosis (Table 4). However, the significance of this association was substantially improved by inclusion of MacReg and IFNγRes status in this model (Table 3, Additional file 1: Table S5).

**Table 2:** The results of univariate Cox PH ratio analysis testing the association of 53 immunogenomic covariates with OS in discovery, validation 1, and pooled cohorts

Variable	Discovery (N = 139)			Validation 1 (N = 70)			Pooled (Discovery + validation 1) (N = 209)		
	ß	HR (95% CI)	P	ß	HR (95% CI)	P	ß	HR (95% CI)	P
*TMB (low)*	*1.26*	*3.52 (2.00–6.20)*	*1.30E–05*	*1.05*	*2.86 (1.23–6.62)*	*0.01*	*1.21*	*3.36 (2.10–5.36)*	*4.01E–07*
*IFNγRes*	*0.46*	*0.63 (0.48–0.84)*	*1.43E–03*	*– 0.76*	*0.47 (0.27–0.82)*	*8.00E–03*	* − 0.50*	*0.60 (0.48–0.77)*	*4.99E–05*
*MacReg*	*0.48*	*0.62 (0.43–0.88)*	*8.18E–03*	*– 0.59*	*0.56 (0.35–0.89)*	*0.02*	* − 0.50*	*0.61 (0.46–0.80)*	*3.87E–04*
Tumor infiltration (regional) fraction	0.05	0.95 (0.91–0.99)	0.02	– 0.04	0.96 (0.90–1.02)	0.19	− 0.05	0.96 (0.92–0.99)	8.00E–03
*Lymphocyte infiltration signature score*	*0.22*	*0.80 (0.67–0.97)*	*2.29E–02*	*– 0.25*	*0.78 (0.62–0.99)*	*0.04*	* − 0.22*	*0.80 (0.70–0.93)*	*2.40E–03*
Number of CNV segments	0.00	1.00 (1.00–1.00)	0.02	3.00E–03	1.00 (1.00–1.01)	0.07	1.74E–03	1.00 (1.00–1.00)	4.00E–03
Activated mast cells	0.22	1.24 (1.01–1.53)	0.04	17.38	3.55E + 7 (7.40E − 07–1.70E + 21)	0.28	11.57	1.06E + 5 (7.54–1.49E + 09)	0.02

Beta values (ß), hazard ratios and 95% confidence intervals (HR 95% CI), and p-values (P) are reported. Significant associations with TMB, age at diagnosis, IFNγRes, MacReg, Lymphocyte-infiltration signature score, and number of CNV segments are in italic

**Table 3:** The 10 most significant multivariate Cox PH models as measured by log-rank p-value

Model (adjusted by age and stage at primary diagnosis)	Discovery (N = 139) P	Validation 2 (N = 69) P	Pooled (Discovery + Validation 2, N = 208) P	Meta (N = 278) P
*TMB* + *MacReg* + *IFNγRes*	*1.70E−07*	*1.28E−02*	*3.37E−10*	*8.80E−14*
TMB + IFNγRes	1.71E−07	1.74E−02	8.26E−10	2.09E−13
MacReg * IFNγRes + TMB	2.16E−07	1.03E−02	1.12E−09	3.30E−13
TMB + Lymphocyte infiltration signature score + IFNγRes	2.84E−07	1.44E−02	6.87E−10	2.88E−13
TMB + MacReg	2.90E−07	6.66E−03	5.23E−10	2.64E−13
Lymphocyte infiltration signature score * IFNγRes + TMB	3.24E−07	2.51E−02	1.97E−09	1.10E−12
TMB * MacReg + IFNγRes	3.40E−07	2.26E−03	6.04E−10	9.59E−14
TMB * IFNγRes + MacReg	3.53E−07	2.22E−02	1.18E−09	3.46E−13
Lymphocyte infiltration signature score * IFNγRes + TMB + MacReg	4.07E−07	3.80E−02	3.05E−09	1.02E−12
TMB * IFNγRes	4.84E−07	3.16E−02	3.04E−09	9.12E−13

Models were adjusted by age and stage at primary diagnosis with covariates that were associated with survival in univariate Cox PH regressions. Interaction effects between variables were assessed (*). The model that was most significantly associated with survival included MacReg, IFNγRes, and TMB. The full table of results are provided in Additional file 1: Table S5

**Table 4:** Multivariate Cox PH model testing the association of nominated variables with melanoma OS

Covariate	N	HR (95% CI)	P
Age at primary diagnosis*	278 (100.0%)	1.02 (1.01–1.04)	7.06E−04
Stage at primary diagnosis			
Local	137 (49.3%)	Ref	
Regional/advanced	141 (50.7%)	1.69 (1.15–2.47)	7.19E−03
TMB			
High	220 (79.1%)	Ref	
Low	58 (20.9%)	2.56 (1.67–3.92)	1.57E−05
MacReg*	278 (100.0%)	0.75 (0.56–1.00)	4.76E−02
IFNγRes*	278 (100.0%)	0.80 (0.63–1.02)	7.16E−02
Log-rank test: P = 8.80E−14			

Nominated variables included low TMB (defined as ≤ 125 mutations), age at diagnosis*, MacReg*, IFNγRes*, and stage at diagnosis (local (stage I/II), regional/advanced (stage III/IV)). Hazard ratios and 95% confidence intervals (HR 95% CI) and p-values (P) for each variable are reported. The overall model goodness of fit as determined by the log-rank test is 8.80E−14

^*^Was used as a continuous variable in Multivariate Cox proportional hazard model

**Fig. 1:**
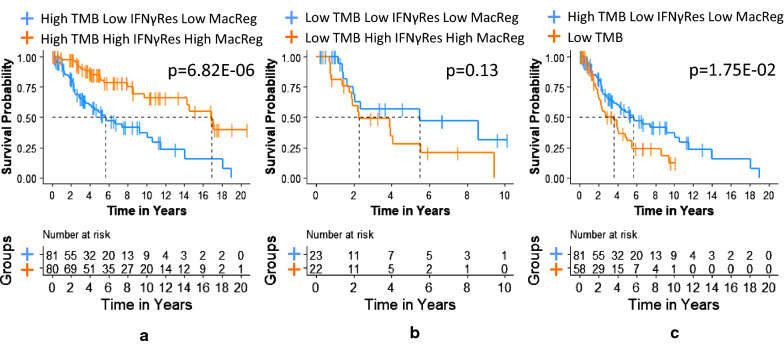
Kaplan–Meier survival curves showing the association of IFNγRes and MacReg signatures in high TMB and low TMB cohorts with melanoma OS. The analysis is performed in high TMB (> 125 mutations) (**a**) or low TMB (≤ 125 mutations) (**b**). Panel A shows high IFNγRes and high MacReg vs low IFNγRes and low MacReg in patients with high TMB, panel B shows high IFNγRes and high MacReg vs low IFNγRes and low MacReg in patients with low TMB and **c** shows low IFNγRes and low MacReg in patients with high TMB vs. patients with low TMB. The vertical dotted lines represent median survival times. All p-values are from log-rank tests

To test this multivariate Cox PH model conditioned on TMB status, we divided the patients into high and low TMB groups (TMB threshold = 125 mutations) to explore whether addition of MacReg and IFNγRes would specifically impact OS in each of the groups, and hence improve prognostic assessment of TMB. The multivariate Cox PH (including MacReg and IFNγRes) was significantly associated with survival in the high TMB (log-rank p = 6.63E−08) but not in the low TMB group (log-rank p = 1.11E−01) (Table 5) indicating that MacReg and IFNγRes both further improved prediction of survival in patients with high TMB. In contrast, in patients with low TMB, the overall Cox PH multivariate model that included age, stage, MacReg and IFNγRes was not significant (Table 5). However, we observed MacReg to have a significant association with OS in the low TMB group (p = 3.43E−02), which was not seen for IFNγRes or other covariates in this model (Table 5). This prompted us to test whether MacReg was specifically associated with OS in low TMB patients in a Cox PH model adjusted for age and stage. We observed that low TMB patients have more favorable survival with increased tumor MacReg (p = 3.26E−02, HR = 0.65, 95% CI 0.436–0.965), but there was no association of OS with IFNγRes, which is in contrast to the association of IFNγRes with OS observed in high TMB patients (Table 5). Based on these observations, we next performed a Kaplan–Meier (KM) analysis in each TMB group separately, by IFNγRes and MacReg status. KM analysis of the high TMB group showed that patients with high levels of MacReg (defined as greater than the overall median value of 0.25) and IFNγRes (greater than the overall median value of – 0.05) had significantly (p = 0.0001) improved survival when compared to those patients with high TMB who had low levels of MacReg (≤ 0.25) and IFNγRes (≤ –0.05) (Fig. 1a). These data clearly show that high TMB patients have different survival outcomes based on their status of MacReg and IFNγRes; the high TMB patients with high levels of MacReg and IFNγRes have significantly better OS compared to those patients with low levels of each, and this association is statistically significant in a univariate Cox PH model (log-rank p = 6.82E−06, HR = 0.31, 95% CI 0.18–0.53) and even more significant in a multivariate Cox PH model (log-rank p = 3.84E−08, HR = 0.35, 95% CI 0.20–0.60). Among low TMB patients, however, no statistical difference in OS was observed between those with high or low levels of MacReg and IFNγRes in either the KM (Fig. 1b) or Cox PH univariate (log-rank p = 0.13) or multivariate analyses (log-rank p = 0.15), suggesting that IFNγRes and MacReg modulate survival only in high TMB and not low TMB patients. Nevertheless, the patients with the least favorable OS among high TMB patients (high TMB, low MacReg and low IFNγRes) had still significantly improved OS compared to those with low TMB regardless of their MacReg and IFNγRes levels (log-rank p = 1.75E−02, HR = 1.75, 95% CI 1.10–2.80) (Fig. 1c) and even more so in a multivariate Cox PH model (log-rank p = 4.71E−04, HR = 1.60, 95% CI 0.99–2.58).

**Table 5:** Multivariate Cox PH models testing the associations of age, stage, MacReg, and IFNγRes with melanoma OS in high TMB and low TMB subpopulations

Model	Covariate	TMB High (N = 220)			TMB Low (N = 58)		
		HR (95% CI)	Covariate P (Wald)	Model P (log-rank)	HR (95% CI)	Covariate P (Wald)	Model P (log-rank)
Age + Stage + MacReg	Age	1.02 (1.01–1.04)	2.17E−03	9.70E−07	1.02 (0.99–1.05)	0.23	0.07
	Stage	1.89 (1.20–2.97)	5.74E−03		1.31 (0.66–2.61)	0.44	
	MacReg	0.64 (0.49–0.84)	1.19E−03		0.65 (0.44–0.97)	3.26E−02	
Age + Stage + IFNγRes	Age	1.03 (1.01–1.04)	6.50E−04	4.40E−08	1.02 (0.99–1.05)	0.21	0.32
	Stage	2.11 (1.34–3.33)	1.36E−03		1.22 (0.61–2.45)	0.57	
	IFNγRes	0.61 (0.48–0.77)	4.39E−05		0.83 (0.58–1.19)	0.31	
Age + Stage + MacReg + IFNγRes	Age	1.02 (1.01–1.04)	1.42E−03	6.60E-08	1.02 (0.99–1.05)	0.20	0.11
	Stage	2.05 (1.30–3.25)	2.06E-03		1.42 (0.69–2.90)	0.34	
	MacReg	0.83 (0.59–1.16)	0.27		0.56 (0.32–0.96)	3.43E-02	
	IFNγRes	0.67 (0.50–0.90)	7.75E-03		1.22 (0.75–1.97)	0.42	

Hazard ratios and 95% confidence intervals (HR 95% CI) and p-values for each variable in each model are reported. Overall model goodness-of-fit as determined by log-rank tests are reported

To test whether TMB, MacReg and IFNγRes also predict post-accession survival of metastatic tumors, we have examined TMB/MacReg/ IFNγRes model using the data of post-accession survival (survival from the time point of advanced tumor collection), which was available for N = 195 patients. Interestingly, we found that the overall multivariate Cox PH model of post-accession survival was statistically significant (log-rank p = 1.94E−09) (Additional file 1: Table S6), confirming that in addition to OS analyses, TMB/MacReg/ IFNγRes associate with survival in metastatic melanoma. While both TMB (log-rank p = 3.87E−07 h = 3.54, 95% CI 2.17–5.77) and MacReg (log-rank p = 1.88E−03, HR = 0.60, 95% CI 0.43–0.83) also showed significant associations in univariate analyses, IFNγRes or age (at time of tumor collection) were not significant in univariate setting.

## Discussion

The role of host immunity in the anti-tumor response has been best documented by the recent successes of ICI treatments in patients with metastatic melanoma. Tumor mutation burden/neo-epitope presentation has been established as a marker of ICI response in melanoma [4, 9, 22] and other cancers [3, 23–25], and has been suggested to be a potential prognostic marker [26, 27], in particular for patients treated with ICI. All this evidence suggests that in tumors with a high tumor mutation burden (TMB) and hence increased likelihood of presentation of neo-epitopes, the immune response may be substantially more effective, leading to increased recruitment of tumor infiltrating lymphocytes (TILs) [28] and significant improvement in treatment outcome. Nevertheless, the paradigm of TMB resulting in a robust anti-tumor response elicited by host immunity has been almost exclusively described in the context of ICI therapies for metastatic disease [3, 4, 9, 22–25]. Recently, large pan-cancer studies based on the TCGA have identified six non-cancer-specific immune subtypes (C1–C6) [11], across different cancers. While this pan-cancer analysis suggested that the level of immune anti-tumor surveillance defined by these six immune subtypes impacted cancer progression, and likely modulates cancer survival, these data have promoted the idea that the immune and tumor repertoire have a concurrent role in cancer progression. In immunogenic tumors, such as melanoma, this provides an excellent opportunity for a melanoma-specific analysis combining TMB, immune signatures, and clinical variables for testing whether the combination of these surrogates can improve personalized assessment of survival, independent of ICI. To date no such detailed analysis quantifying the specific effects of the immunogenomic component interaction on melanoma OS has been performed. In our study, we first tested whether TMB/neo-epitope presentation observed in metastatic melanoma may be associated with prolonged patient survival, independent of ICI. In conjunction with immune-related factors determined by the recent TCGA pan-cancer analysis, we further explored whether TMB can be integrated with other immune and tumor-specific correlates to improve current prognostic models of melanoma survival. In a sample of 278 metastatic tumors from melanoma patients from publicly available resources at TCGA who had not been previously treated by ICI therapies, we found that low TMB was associated with worse OS (discovery p = 1.30E−05, HR = 3.52, 95% CI 2.00–6.20 and validation 1 p = 0.01, HR = 2.86, 95% CI 1.23–6.62). These data suggest that TMB, in patients not treated by ICI, may be a surrogate for tumor immunogenicity, thus impacting OS. Specifically, our findings show that melanoma OS is significantly prolonged in patients with metastatic tumors harboring > 125 mutations, a threshold that is consistent with prior studies focused on ICI outcomes: a meta-analysis of three ICI metastatic melanoma studies [9] determined that 192 mutations best differentiated responses to ICI therapy. In another study, with a much smaller sample size, a low mutation burden (≤ 100 mutations) was associated with ICI resistance and worse progression-free survival [4]. In NSCLC, Rizvi et al. [3] found similar results with response to ICI therapy by defining low and high TMB by the median of 209 mutations and found that the high mutation load was associated with both improved progression-free survival and response to ICI therapy. Again, while this threshold is higher than the TMB cut-off producing the most significant association in our study (N = 125 mutations), we showed a positive association between TMB and OS using a threshold of ≤ 200 mutations (Table 1). This indicates that TMB can act consistently in both melanoma and NSCLC as a predictor of OS. Importantly, our findings also show that TMB can be a surrogate for NB, which in our data displayed a high degree of correlation (Pearson’s r = 0.979, p < 1E−20) (Additional file 1: Figure S2). In contrast to a previous report [4] in which the calculation of NB was necessary to find an association with ICI therapy outcomes, in our data, TMB was a more robust predictor of OS compared to NB (Table 1 and Additional file 1: Table S3). This suggests that TMB can be a reliable prognostic indicator of melanoma OS, independently of ICI or presentation of other histological and clinical characteristics. We have further capitalized on data available from a recent pan-cancer immune landscape TCGA study [11] to evaluate how the prognostic value of TMB can be enhanced by the addition of immunogenomic features from tumors of metastatic melanoma patients. We tested 54 features (53 immunogenomic characteristics from Thorsson et al. [11] and TMB) and identified four features that were significantly associated with survival in both the discovery and validation patient cohorts (Table 2). Besides TMB, these included the immune expression signatures of IFNγRes, MacReg, and Lymphocyte infiltration. In our data, however, the most significant prognostic Cox PH models were all combinations of TMB with other immunogenomic markers; such TMB-containing Cox PH models were much more significant than TMB alone and more significant than any other immunogenomic features tested individually or in combination (Table 3). The most significant Cox PH model in our entire study was the combination of TMB with MacReg and IFNγRes levels (log-rank p = 8.80E−14) (Table 3), adjusted by age and stage. These data suggest that the immune surveillance of tumor progression in melanoma is impacted by an interplay of TMB and other immune characteristics of the TME, which in turn collectively affect patient survival from early tumor stages. This is shown by our findings that IFNγRes and MacReg levels modulate survival differently in patients with high or low TMB (Fig. 1). We found that the levels of both MacReg and IFNγRes levels stratified patients with high TMB into two distinct survival groups; decreased levels of MacReg and IFNγRes in TMB high patients were associated with significantly worse survival, compared to high levels of both MacReg and IFNγRes. This shows that among patients with high TMB, which has been considered to be a favorable prognostic indicator, there is further stratification of survival based on the status of other pro-inflammatory tumor intrinsic pathways. These data have significant clinical potential, as they further refine currently established prognostic paradigms, supporting the need for the assessment of the levels of MacReg and IFNγRes in melanoma prognostic algorithms. Our data (Table 5) also show that while both MacReg and IFNγRes contribute to the association with OS in high TMB patient groups, it is actually IFNγRes in the high TMB group that most significantly drives this association. In the most significant overall model in the high TMB group (log-rank p = 6.6E−08) (Table 5) that includes the IFNγRes, the effect of MacReg disappears. In contrast, this OS model (including age, stage, MacReg and IFNγRes) in the low TMB group was not significant. There was, however, some significance observed for the association with OS in the low TMB group for MacReg regulation, but not IFNγRes. In general, and as also shown by our data, there is a correlation between both of these pro-inflammatory signaling pathways (MacReg and IFNγRes), whose upregulation is likely associated with an immunostimulatory environment. This is in accordance with the established model of the activation of macrophages in the anti-tumor immune response, often mediated by IFNγRes signaling. However, the opposite effects for the increased levels of IFNγRes (associated with OS in the TMB high group) and MacReg (associated with OS in the TMB low group), suggest different biological mechanisms of anti-tumor immunity in tumors with low TMB, likely supporting a model of a direct stimulation of MacReg that is independent of IFNγRes signaling. Upregulation of MacReg may be mediated through other co-stimulatory pathways, such as activation by granulocytes [29], Th2 cells [30], or glycoproteins [31], many of which have been shown to associate with improved anticancer immune response [32]. This may represent an alternative mechanism of immune stimulation in TMB-low melanomas with increased levels of MacReg signaling. While this may have significant biological and prognostic implications, it is important to note that the TMB-low group in our data was underpowered (only 58 patients with low TMB), and the interpretation of these findings will need independent validation. In general, the findings of this study can be strengthened by further validation in additional datasets with well harmonized clinical information on patients not previously treated by ICI, and with the availability of significant follow up for prognostic assessment. However, the availability of such datasets with gene expression profiles from tumor RNA sequencing, tumor whole exome sequencing data, and sufficient survival data from time of primary diagnosis, is presently limited. With the reduction of sequencing cost, allowing for a comprehensive buildup of large repositories of personalized genomes and transcriptomes for a large number of melanoma patients with well-harmonized clinical follow up information, the validation of current findings will become feasible in the foreseeable future. Importantly, the retrospective analysis of completed clinical trials (such as early generation of immunotherapy treatments, e.g. IL2, INF gamma, or placebo arms of immune checkpoint inhibition) may provide additional support for prognostic clinical biomarker validity of these findings. Another limitation of our study includes the focus on retrospective prediction of survival from early stages based on TMB in metastatic tumors. From a clinical perspective, the immunogenomic landscape as a biomarker of survival will need to be explored in primary melanoma tissues with sufficient follow-up survival information. While there are melanoma primary tissues in the TCGA skin cancer dataset (N = 107), a major roadblock is that the survival time information for these primary melanomas is insufficient. In contrast, the advanced tumors used in our study had extensive retrospective information available on survival follow-up from primary diagnosis allowing for the assessment of immunogenomic features impacting OS. Nevertheless, it is important to underscore that the prognostic assessment based on indicators from more advanced tumors may have a specific clinical value for prediction of survival of patients with advanced disease not treated by ICI, as we demonstrated by the analysis of post-accession data (from the time of tumor collection until death or last follow up). This important analysis showed that the same Cox model of TMB/IFNγRes/MacReg is highly significant in the association analysis of survival of patients with advanced disease (log-rank p = 1.94E−09) (Additional file 1: Table S6). Hence, our findings, while generated using the data from advanced tumors, serve as an important indicator that TMB combined with other immunogenomic tumor characteristics may refine prognostic assessment of melanoma survival.

## Conclusions

In summary, to our knowledge this is the first study that highlights and systematically quantifies the contribution and the effect of TMB and other immunogenomic features from the TME on OS in melanoma patients not previously treated by ICI. We developed a model combining TMB, IFNγRes and MacReg immune expression signatures, stage and age at diagnosis that significantly improves prediction of melanoma OS in patients not treated by ICI. We showed that decreased IFNγRes and MacReg signaling identifies a group of high TMB patients with less favorable prognosis, who may benefit from more targeted clinical prognostic management.

Upon further validation of these findings in primary tumors with extensive and sufficient long-term follow-up, the data presented here may significantly refine current prognostic algorithms complementing the established AJCC-based prognostic assessment, potentially revealing novel biological mechanisms of melanoma progression controlled by host immunity.

## Supplementary Information

**Additional file 1. **Supplementary Tables and Figures: **Table S1**. Immunogenomic and tumor microenvironment characteristics of TCGA SKCM patients; **Table S2**. Patient characteristics in the study population (Discovery, Validation 1 and Validation 2); **Table S3**. Neo-epitope thresholds derived from TMB; **Table S4**. Cox proportional hazards assumption verification; **Table S5**. Multivariate Cox PH models; **Table S6**. Multivariate Cox PH regression showing the association of covariates and post-accession survival; **Figure S1**. Kaplan-Meier survival curves showing the association of tumor mutation burden with melanoma survival; **Figure S2**. Scatterplot showing relationship between tumor mutation burden and neo-epitope burden.

## Data Availability

Clinical, demographic, and tumor biospecimen data, including WXS data of tumor tissues in BAM format, are available to download via the TCGA GDCDP. Additional data analyzed in this study, including immunogenomic signatures and histological data, are provided by Thorsson et al. [11].

## Abbreviations

AJCC: American Joint Committee for Cancer
CI: Confidence interval
Cox PH: Cox proportional-hazards
GDCDP: Genomic Data Commons Data Portal
KM: Kaplan–Meier
HR: Hazard ratio
ICI: Immune checkpoint inhibition
IFNγRes: Interferon-gamma response
IT: Immunotherapy
MacReg: Macrophage regulation
NB: Neo-epitope burden
NSCLC: Non-small cell lung cancer
OS: Overall survival
SKCM: Skin cutaneous melanoma
TCGA: The Cancer Genome Atlas
TMB: Tumor mutation burden
TME: Tumor microenvironment
WXS: Whole-exome sequencing
