# Correction: Tumor immunogenomic signatures improve a prognostic model of melanoma survival

**DOI:** 10.1186/s12967-023-04040-7

**Published:** 2023-03-31

**Authors:** Leah Morales, Danny Simpson, Robert Ferguson, John Cadley, Eduardo Esteva, Kelsey Monson, Vylyny Chat, Carlos Martinez, Jefrey Weber, Iman Osman, Tomas Kirchhof

**Affiliations:** 1 Laura and Isaac Perlmutter Cancer Center, NYU Langone Health, 522 First Avenue, New York City, NY 10016 USA; 2 Departments of Population Health and Environmental Medicine, NYU Langone Health, New York, USA; 3 The Interdisciplinary Melanoma Cooperative Group, NYU Langone Health, New York, USA; 4 Department of Medicine, NYU Langone Health, New York, USA; 5 Ronald O. Perelman Department of Dermatology, NYU Langone Health, New York, USA

**Correction: J Transl Med (2021) 19:78** **https://doi.org/10.1186/s12967-021-02738-0**

Following publication of the original article [1], we have been notified that the Funding note was published incorrectly. It should be as follows:

**Funding note**

 This work was supported by a Grant from the National Cancer Institute: R01CA187060-05 and NYU Melanoma SPORE P50CA225450.

## References

[1] Morales L, Simpson D, Ferguson R, Cadley J, Esteva E, Monson K, Chat V, Martinez C, Weber J, Osman I, Kirchhoff T (2021). Tumor immunogenomic signatures improve a prognostic model of melanoma survival. J Transl Med.

