# Supplementary material for: Tumor immunogenomic signatures improve a prognostic model of melanoma survival
Source: J Transl Med. 2021 Feb 17;19:78. doi: 10.1186/s12967-021-02738-0 (PMC7888085; doi:10.1186/s12967-021-02738-0)
Supplement: Supplementary file 1 — Additional file 1. Supplementary Tables and Figures: Table S1. Immunogenomic and tumor microenvironment characteristics of TCGA SKCM patients; Table S2. Patient characteristics in the study population (Discovery, Validation 1 and Validation 2); Table S3. Neo-epitope thresholds derived from TMB; Table S4. Cox proportional hazards assumption verification; Table S5. Multivariate Cox PH models; Table S6. Multivariate Cox PH regression showing the association of covariates and post-accession survival; Figure S1. Kaplan-Meier survival curves showing the association of tumor mutation burden with melanoma survival; Figure S2. Scatterplot showing relationship between tumor mutation burden and neo-epitope burden. [file 12967_2021_2738_MOESM1_ESM.docx]

**Additional data**

| **Variable (Thorsson et al.)** |
| --- |
| Leukocyte Fraction |
| Stromal Fraction |
| Intratumor Heterogeneity |
| TIL Regional Fraction |
| Proliferation |
| Wound Healing |
| Macrophage Regulation |
| Lymphocyte Infiltration Signature Score |
| IFN-gamma Response |
| TGF-beta Response |
| Silent Mutation Rate |
| Nonsilent Mutation Rate |
| Number of Segments |
| Fraction Altered |
| Aneuploidy Score |
| Homologous Recombination Defects |
| BCR Evenness |
| BCR Shannon |
| BCR Richness |
| TCR Shannon |
| TCR Richness |
| TCR Evenness |
| Th1 Cells |
| Th2 Cells |
| Th17 Cells |
| B Cells Memory |
| B Cells Naive |
| Dendritic Cells Activated |
| Dendritic Cells Resting |
| Eosinophils |
| Macrophages M0 |
| Macrophages M1 |
| Macrophages M2 |
| Mast Cells Activated |
| Mast Cells Resting |
| Monocytes |
| Neutrophils |
| NK Cells Activated |
| NK Cells Resting |
| Plasma Cells |
| T Cells CD4 Memory Activated |
| T Cells CD4 Memory Resting |
| T Cells CD4 Naive |
| T Cells CD8 |
| T Cells Follicular Helper |
| T Cells gamma delta |
| T Cells Regulatory Tregs |
| Lymphocytes |
| Neutrophils |
| Eosinophils |
| Mast Cells |
| Dendritic Cells |
| Macrophages |

Table S1. Fifty-three immunogenomic and tumor microenvironment characteristics of TCGA SKCM patients as provided by Thorsson et al.^1^ evaluated for association with melanoma OS.

|  | **Discovery** | **Validation 1** | **Validation 2** | **Total Population (Meta)** | **Association with survival in Meta (P)** |
| --- | --- | --- | --- | --- | --- |
|  | **(N = 139)** | **(N = 70)** | **(N = 69)** | **(N = 278)** |  |
| **Age at diagnosis (median)** | 57 | 56 | 60 | 58 | 5.08E-05 |
| **Sex (number of males)** | 90 (64.7%) | 44 (62.9%) | 47 (68.1%) | 181 (65.1%) | 0.93 |
| **Stage** |  |  |  |  | 3.22E-03 |
| **Local** | 57 (41.0%) | 40 (57.1%) | 40 (57.1%) | 137 (49.3%) |  |
| **Regional/advanced** | 82 (59.0%) | 30 (42.9%) | 29 (42%) | 141 (50.7%) |  |
| **Treatment received** |  |  |  |  |  |
| **Patients treated with radiation** | 43 (30.9%) | 28 (40.0%) | 14 (20.3%) | 85 (30.6%) | 0.79 |
| **Patients treated with BRAF/MEKi** | 5 (3.6%) | 0 (0%) | 3 (4.4%) | 8 (2.9%) | 0.77 |

Table S2. Patient characteristics in the study population including the breakdown of the patients in the Discovery, Validation 1 and Validation 2 groups. Age at diagnosis is given by its median value. Univariate Cox PH was used to calculate the association of patient characteristics with OS.

|  |  | **Discovery (N = 139)** | |  | **Validation 1 (N = 70)** | |  | **Pooled (Discovery + Validation 1) (N = 209)** | |
| --- | --- | --- | --- | --- | --- | --- | --- | --- | --- |
| **Threshold** | **N < Threshold** | **P** | **HR (95% CI)** |  | **P** | **HR (95% CI)** |  | **P** | **HR (95% CI)** |
| 25 | 19 (13.7%) | 4.06E-04 | 3.45 (1.74 - 6.87) |  |  |  |  |  |  |
| **50** | **30 (21.6%)** | **3.84E-05** | **3.50 (1.93 - 6.35)** |  | **0.165** | **1.78 (0.79- 4.01)** |  | **5.60E-05** | **2.64 (1.65- 4.23)** |
| 75 | 38 (27.3%) | 8.07E-04 | 2.54 (1.47 - 4.40) |  |  |  |  |  |  |
| 100 | 47 (33.8%) | 1.79E-02 | 1.89 (1.12 - 3.21) |  |  |  |  |  |  |
| 125 | 52 (37.4%) | 1.67E-02 | 1.90 (1.12 - 3.21) |  |  |  |  |  |  |
| 150 | 58 (41.7%) | 8.10E-03 | 2.03 (1.2 - 3.42) |  |  |  |  |  |  |
| 189 | 70 (50.4%) | 6.23E-02 | 1.65 (0.97 – 2.79) |  |  |  |  |  |  |

Table S3. The different thresholds of the number of neo-epitopes derived from TMB, using a POLYSOLVER/netMHC pipeline, defining high and low NB, and the corresponding number of low NB patients per each threshold (N < Threshold) in the discovery phase. The Wald p-values (P), hazard ratios and associated 95% confidence intervals (HR 95% CI) for each threshold in the discovery phase were derived from univariate Cox PH models. The 50 NB threshold (bolded), with the most significant p-value, was moved forward to the validation stage 1. The Wald p-value (P) from the univariate Cox PH analysis at the validation stage are also included, along with the associated hazard ratio and 95% confidence interval (HR 95% CI). The final two columns give the Wald p-value (P) for the pooled-analysis of both the discovery and validation population, with accompanying hazard ratio and 95% confidence interval (HR 95% CI) from a univariate Cox PH model.

| **Covariate** | **Rho** | **P** |
| --- | --- | --- |
| Age at diagnosis | 0.4370 | 0.5085 |
| Stage (regional/advanced) | -0.1448 | 0.1161 |
| TMB (low) | 0.0515 | 0.5488 |
| Interferon-γ response | 0.1602 | 0.0753 |
| Macrophage regulation | -0.0353 | 0.6770 |
| GLOBAL |  | 0.2523 |

Table S4. The Cox proportional hazards assumption was verified for all covariates in the multivariate Cox PH model applied to all patients (meta) testing for independence of the scaled Schoenfeld residuals with time. Rho indicates the Pearson correlation between the scaled Schoenfeld residuals and time, along with the associated p-value (P). A global chi-squared test was performed using all covariates and again, showed no association. As no covariates showed an association with time, the proportional hazards assumption was verified in all cases.

| Model (adjusted by age and stage at primary diagnosis) | Discovery (N=139) | Validation 2 (N=69) | Pooled (Discovery + Validation 2, N=208) | Meta (N=278) |
| --- | --- | --- | --- | --- |
|  | P | P | P | P |
| **TMB + MacReg + IFNγRes** | **1.70E-07** | **1.28E-02** | **3.37E-10** | **8.80E-14** |
| TMB + IFNγRes | 1.71E-07 | 1.74E-02 | 8.26E-10 | 2.09E-13 |
| MacReg * IFNγRes + TMB | 2.16E-07 | 1.03E-02 | 1.12E-09 | 3.30E-13 |
| TMB + Lymphocyte infiltration signature score + IFNγRes | 2.84E-07 | 1.44E-02 | 6.87E-10 | 2.88E-13 |
| TMB + MacReg | 2.90E-07 | 6.66E-03 | 5.23E-10 | 2.64E-13 |
| Lymphocyte infiltration signature score * IFNγRes + TMB | 3.24E-07 | 2.51E-02 | 1.97E-09 | 1.10E-12 |
| TMB * MacReg + IFNγRes | 3.40E-07 | 2.26E-03 | 6.04E-10 | 9.59E-14 |
| TMB * IFNgRes + MacReg | 3.53E-07 | 2.22E-02 | 1.18E-09 | 3.46E-13 |
| Lymphocyte infiltration signature score * IFNγRes + TMB + MacReg | 4.07E-07 | 3.80E-02 | 3.05E-09 | 1.02E-12 |
| TMB * IFNγRes | 4.84E-07 | 3.16E-02 | 3.04E-09 | 9.12E-13 |
| TMB + Lymphocyte infiltration signature score + MacReg + IFNγRes | 5.08E-07 | 2.44E-02 | 1.18E-09 | 2.92E-13 |
| TMB + Lymphocyte infiltration signature score | 5.55E-07 | 8.24E-03 | 1.54E-09 | 2.09E-12 |
| MacReg * IFNγRes + TMB + Lymphocyte infiltration signature score | 5.86E-07 | 1.89E-02 | 3.52E-09 | 9.79E-13 |
| TMB * IFNγRes + Lymphocyte infiltration signature score | 6.17E-07 | 2.23E-02 | 2.34E-09 | 1.13E-12 |
| TMB * Lymphocyte infiltration signature score * IFNγRes | 7.69E-07 | 2.94E-04 | 6.28E-10 | 3.87E-13 |
| TMB * MacReg + Lymphocyte infiltration signature score * IFNγRes | 7.80E-07 | 8.12E-03 | 4.10E-09 | 8.52E-13 |
| TMB * MacReg | 7.81E-07 | 1.12E-03 | 8.09E-10 | 2.32E-13 |
| TMB * Lymphocyte infiltration signature score + IFNγRes | 8.07E-07 | 3.04E-04 | 3.97E-10 | 1.38E-13 |
| TMB * Lymphocyte infiltration signature score * IFNγRes + MacReg | 8.78E-07 | 2.99E-04 | 4.74E-10 | 1.60E-13 |
| TMB + Lymphocyte infiltration signature score + MacReg | 9.18E-07 | 1.41E-02 | 1.93E-09 | 1.10E-12 |
| TMB * MacReg + Lymphocyte infiltration signature score + IFNγRes | 9.47E-07 | 4.52E-03 | 1.93E-09 | 2.79E-13 |
| TMB * IFNγRes + Lymphocyte infiltration signature score + MacReg | 9.77E-07 | 3.74E-02 | 3.71E-09 | 1.03E-12 |
| Lymphocyte infiltration signature score * MacReg + TMB + IFNγRes | 1.31E-06 | 4.27E-02 | 3.69E-09 | 1.01E-12 |
| TMB * Lymphocyte infiltration signature score + MacReg + IFNγRes | 1.34E-06 | 5.95E-04 | 6.53E-10 | 1.34E-13 |
| TMB * Lymphocyte infiltration signature score + MacReg * IFNγRes | 1.45E-06 | 9.95E-04 | 1.93E-09 | 4.45E-13 |
| TMB * Lymphocyte infiltration signature score | 1.76E-06 | 2.23E-04 | 8.86E-10 | 9.72E-13 |
| TMB * MacReg * IFNγRes | 2.05E-06 | 3.18E-03 | 5.38E-09 | 5.91E-13 |
| TMB * MacReg + Lymphocyte infiltration signature score | 2.13E-06 | 2.46E-03 | 2.73E-09 | 8.44E-13 |
| TMB * IFNγRes + Lymphocyte infiltration signature score * MacReg | 2.42E-06 | 6.07E-02 | 1.07E-08 | 3.33E-12 |
| Lymphocyte infiltration signature score * MacReg + TMB | 2.57E-06 | 2.70E-02 | 6.48E-09 | 3.88E-12 |
| TMB * Lymphocyte infiltration signature score + MacReg | 2.64E-06 | 3.99E-04 | 1.06E-09 | 4.92E-13 |
| TMB * Lymphocyte infiltration signature score * MacReg * IFNγRes | 3.56E-06 | 9.31E-05 | 1.39E-08 | 4.68E-12 |
| TMB | 3.77E-06 | 2.86E-02 | 1.09E-07 | 1.43E-10 |
| TMB * MacReg * IFNγRes + Lymphocyte infiltration signature score | 4.71E-06 | 4.83E-03 | 1.32E-08 | 1.12E-12 |
| Lymphocyte infiltration signature score * MacReg * IFNγRes + TMB | 4.87E-06 | 1.17E-02 | 1.40E-08 | 1.18E-11 |
| TMB * Lymphocyte infiltration signature score * MacReg + IFNγRes | 5.47E-06 | 3.76E-04 | 4.54E-09 | 1.90E-12 |
| TMB * Lymphocyte infiltration signature score * MacReg | 1.56E-05 | 3.42E-04 | 1.22E-08 | 7.34E-12 |
| IFNγRes | 1.71E-05 | 1.93E-02 | 3.69E-07 | 1.80E-09 |
| MacReg * IFNγRes | 2.03E-05 | 2.61E-02 | 1.07E-06 | 7.45E-09 |
| MacReg + IFNγRes | 2.07E-05 | 1.93E-02 | 3.31E-07 | 2.05E-09 |
| Lymphocyte infiltration signature score * IFNγRes + MacReg | 2.45E-05 | 6.31E-02 | 1.83E-06 | 1.07E-08 |
| Lymphocyte infiltration signature score * IFNγRes | 3.59E-05 | 4.48E-02 | 1.63E-06 | 1.64E-08 |
| MacReg * IFNgRes + Lymphocyte infiltration signature score | 3.85E-05 | 4.50E-02 | 2.68E-06 | 1.34E-08 |
| Lymphocyte infiltration signature score + IFNγRes | 4.25E-05 | 2.40E-02 | 7.39E-07 | 5.55E-09 |
| Lymphocyte infiltration signature score + MacReg + IFNγRes | 4.61E-05 | 3.82E-02 | 9.27E-07 | 4.06E-09 |
| Lymphocyte infiltration signature score * MacReg + IFNγRes | 1.01E-04 | 6.35E-02 | 2.51E-06 | 8.33E-09 |
| MacReg | 1.10E-04 | 9.16E-03 | 1.06E-06 | 1.59E-08 |
| Lymphocyte infiltration signature score * MacReg * IFNγRes | 2.53E-04 | 2.19E-02 | 8.00E-06 | 9.60E-08 |
| Lymphocyte infiltration signature score + MacReg | 3.22E-04 | 2.11E-02 | 3.70E-06 | 4.74E-08 |
| Lymphocyte infiltration signature score | 4.16E-04 | 1.29E-02 | 4.95E-06 | 1.61E-07 |
| Lymphocyte infiltration signature score * MacReg | 7.46E-04 | 3.83E-02 | 1.02E-05 | 8.53E-08 |

Table S5. Ranked multivariate Cox PH models ordered by log-rank p-value. Models were adjusted by age and stage at primary diagnosis with covariates that were associated with survival in univariate Cox PH regressions. Interaction effects between variables were assessed (*). The model that was most significantly associated with survival included MacReg, IFNγRes, and TMB.

| **Covariate** | **N** | **HR (95% CI)** | **P** |
| --- | --- | --- | --- |
| Age at accession | 278 (100.0%) | 1.00 (0.98 - 1.02) | 0.99 |
| Tumor mutation burden |  |  |  |
| High | 220 (79.1%) | Ref |  |
| Low | 58 (20.9%) | 3.54 (2.17 - 5.77) | 3.87E-07 |
| MacReg | 278 (100.0%) | 0.60 (0.43 - 0.83) | 1.88E-03 |
| IFNγRes | 278 (100.0%) | 0.83 (0.63 - 1.09) | 0.175 |
|  |  |  |  |
| Log-rank test: P = 1.94E-09 |  |  |  |

Table S6. The multivariate Cox PH regression (originally optimized for OS) showing the association of age at tumor accession, TMB, MacReg, and IFNγRes with post-accession survival (N=195). Patient staging is not included, as all tumors at accession were metastatic. Log-rank p-value for the overall model is provided.


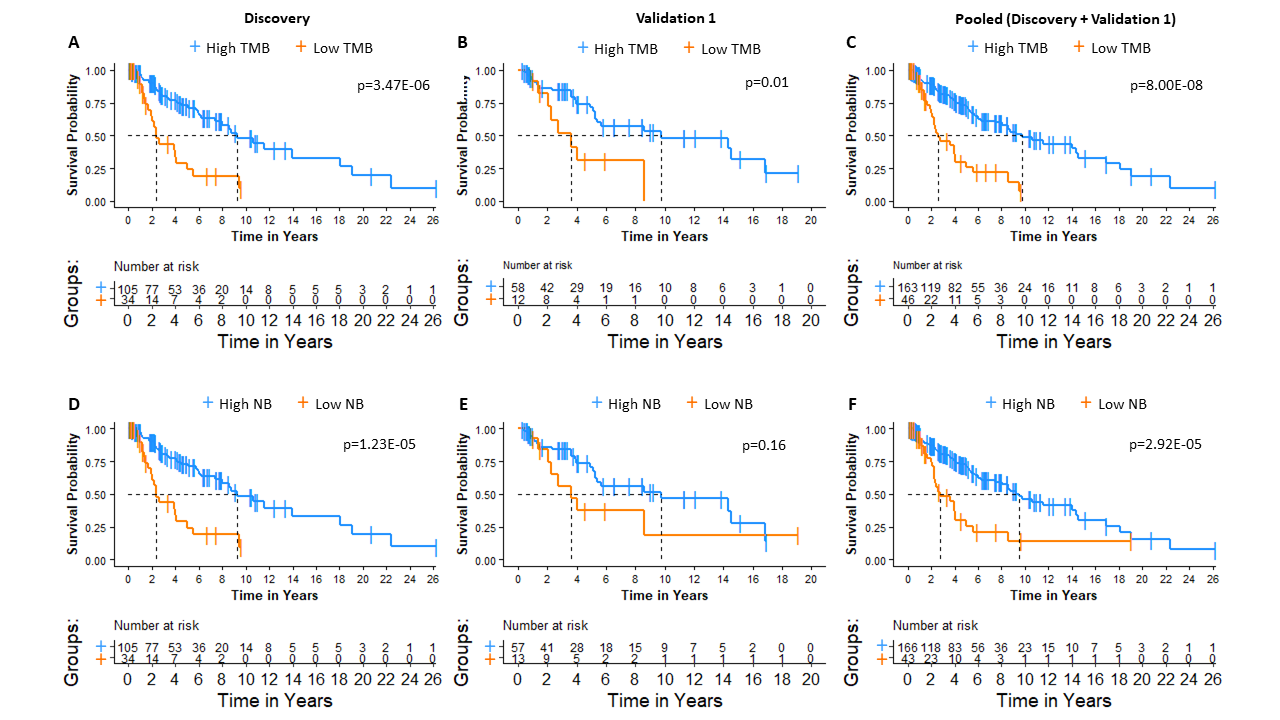


Figure S1. Kaplan-Meier survival curves showing the association of tumor mutation burden with melanoma survival. The analysis is stratified by high TMB (> 125 mutations) or low TMB (≤ 125 mutations) (A, B, C) and by high NB (>50 neo-epitopes) or low NB (≤50 neo-epitopes) (D, E, F). Patients with high TMB show significantly better median overall survival compared to low TMB in the Discovery group (9.3 vs. 2.4 years; log-rank p = 3.47E-06) (A), Validation 1 group (9.8 vs. 3.6 years; log-rank p = 0.01) (B), and the pooled (Discovery + Validation 1) group (9.8vs. 2.6 years; log-rank p =8.00E-08). Significantly improved median survival was also found in patients with high NB compared to low NB in the Discovery (9.3 vs. 2.4 years; log-rank p = 1.23E-05) (D), Validation 1 (E), and pooled (9.5 vs. 2.7 years; log-rank p = 2.91E-06) (F) cohorts.

Figure S2. Scatterplot showing relationship between tumor mutation burden (x-axis) and neo-epitope burden (y-axis) for all individuals in the dataset. TMB and NB show an extremely strong correlation (Pearson’s r=0.979; p<1E-20), indicating that TMB can act as a surrogate for NB.

**REFERENCE**

1 Thorsson, V. *et al.* The Immune Landscape of Cancer. *Immunity* **48**, 812-830 e814, doi:10.1016/j.immuni.2018.03.023 (2018).
